# Multi-institutional study of mesonephric-like adenocarcinoma: its clinicopathological characteristics and histogenetic association with ectopic endometrium

**DOI:** 10.1007/s00428-025-04286-0

**Published:** 2025-10-29

**Authors:** Yu Miyama, Kousuke Uranishi, Masataka Hirasaki, Akiko Tonooka, Fumi Kawakami, Yoshiki Mikami, Takako Kiyokawa, Hiroshi Kajiwara, Junichi Shiraishi, Eiji Kudo, Kozue Ito, Morihiro Higashi, Sachiko Minamiguchi, Nasa Okazaki, Yuzuru Kondo, Tetsuya Shimada, Yoshinobu Maeda, Yumiko Yasuhara, Yoshiaki Kawano, Yohei Kawasaki, Kosei Hasegawa, Masanori Yasuda

**Affiliations:** 1 Department of Pathology, Saitama Medical University International Medical Center, Yamane 1397-1,, Hidaka, 350-1298 Saitama Japan; 2 Division of Biomedical Sciences, Research Center for Genomic Medicine, Saitama Medical University, Hidaka, Saitama Japan; 3 Department of Clinical Cancer Genomics, Saitama Medical University International Medical Center, Hidaka, Saitama Japan; 4 Division of Pathology, Cancer Institute, Japanese Foundation for Cancer Research, Koto, Tokyo Japan; 5 Department of Pathology, Cancer Institute Hospital, Japanese Foundation for Cancer Research, Koto, Tokyo Japan; 6 Department of Diagnostic Pathology, Kumamoto University Hospital, Kumamoto, Kumamoto Japan; 7 Department of Pathology and Cell Biology, University of the Ryukyus, Ginowan, Okinawa Japan; 8 Department of Pathology, The Jikei University School of Medicine, Minato, Tokyo Japan; 9 Department of Pathology, Tokai University School of Medicine, Isehara, Kanagawa Japan; 10 Department of Pathology, National Hospital Organization Tokyo Medical Center, Meguro, Tokyo Japan; 11 Department of Pathology, Tokushima Prefectural Central Hospital, Tokushima, Tokushima Japan; 12 Department of Pathology, Saitama Medical Center, Saitama Medical University, Kawagoe, Saitama Japan; 13 Department of Pathology, Uonuma Kikan Hospital, Minamiuonuma, Niigata Japan; 14 Department of Diagnostic Pathology, Kyoto University Hospital, Kyoto, Kyoto Japan; 15 Department of Diagnostic Pathology, Japanese Red Cross Kumamoto Hospital, Kumamoto, Kumamoto Japan; 16 Department of Pathology, National Hospital Organization Kasumigaura Medical Center, Tsuchiura, Ibaraki Japan; 17 National Hospital Organization Nishisaitama-Chuo National Hospital, Tokorozawa, Saitama Japan; 18 Department of Diagnostic Pathology, Toyama Red Cross Hospital, Toyama, Toyama Japan; 19 Department of Pathology, Sakai City Medical Center, Sakai, Osaka Japan; 20 Department of Obstetrics and Gynecology, Japan Community Healthcare Organization Kyushu Hospital, Kitakyushu, Fukuoka Japan; 21 Department of Biostatistics, Graduate School of Medicine, Saitama Medical University, Iruma-Gun, Saitama Japan; 22 Department of Gynecologic Oncology, Saitama Medical University International Medical Center, Hidaka, Saitama Japan

**Keywords:** Mesonephric-like adenocarcinoma, Endometriosis, Adenomyosis, *KRAS* mutations

## Abstract

To explore the clinicopathological features and origin of mesonephric-like adenocarcinomas (MLAs), 83 cases diagnosed or suspected to be MLAs were collected from various institutions in Japan. We clearly classified 78 as MLAs (uterus: 47, ovary: 31) and 5 as non-MLAs (all ovary) based on our morphological and immunohistochemical criteria. In uterine MLAs (uMLAs), lymphovascular space invasion was an independent prognostic factor for progression-free survival (PFS) (*P* = 0.03). Patients with uMLAs had significantly shorter PFS and overall survival (OS) than those diagnosed with endometrial endometrioid carcinomas (EECs) (*P* < 0.0001 and *P* < 0.001, respectively) and comparable PFS and OS to those with copy number-high tumors. PFS and OS of ovarian MLAs (oMLAs) were similar to those of ovarian endometrioid carcinomas (OECs) overall but worse for patients with stage II-IV. Endometriosis was observed with oMLAs as often as with OECs (94% vs 93%, respectively). Adenomyosis was more frequently observed with uMLAs than with EECs (62% vs 28%, respectively, *P* = 0.0005). Six uMLAs were confined to the myometrium and adjacent to adenomyosis. In an analysis of molecularly speculated origin, among 29 oMLAs harboring a *KRAS* hotspot mutation, 23 (79%) instances of endometriosis in the background had the same mutation. Among 36 uMLAs carrying the *KRAS* hotspot mutation, common mutations were observed in 12 (33%) instances of adjacent adenomyosis (12/21 [67%] of adenomyosis). These findings suggest a histogenetic link between MLAs and ectopic endometrium, implicating both ovarian endometriosis and adenomyosis in MLA pathogenesis, with potential clinical significance.

**Supplementary Information:**

The online version contains supplementary material available at 10.1007/s00428-025-04286-0.

## Introduction

Mesonephric-like adenocarcinomas (MLAs) originate in the female genital system, including the uterus or ovary, and may also arise in the peritoneal cavity. [1–4] First reported by McFarland in 2016 [1] and officially recognized as a distinct entity in the 2020 World Health Organization classification, [5] awareness of MLAs has increased, leading to more frequent diagnoses. They are defined as adenocarcinomas displaying a mesonephric-like phenotype with tubular, glandular, papillary, and solid pattern morphology. Immunohistochemically, they are typically positive for GATA3 and/or TTF1 and negative for estrogen receptor (ER) and progesterone receptor (PR); they also often express luminal CD10 and occasionally calretinin [6, 7].

Recently, grey-zone tumors that are morphologically and immunohistochemically reminiscent of MLA, but cannot entirely be excluded as other histotypes, have been identified. This diagnostic overlap highlights the importance of distinguishing MLA from other histotypes, such as endometrioid carcinoma (EC), given the differences in prognosis. Ovarian (oMLAs) and uterine (uMLAs) MLAs exhibit clinically aggressive behavior [2, 3, 8]. However, in our recent study, we found that the International Federation of Gynecology and Obstetrics (FIGO) I ovarian endometrioid carcinomas (OECs) with mesonephric-like differentiation (MLD) do not differ from conventional OEC regarding prognosis [9].

MLAs are presumed to be of Mullerian origin, and several studies have reported ovarian or extra-ovarian MLAs associated with endometriosis [4, 8, 10]. Based on our own experience, we have encountered cases of uMLA coexisting with adenomyosis, including tumors localized entirely within the myometrium. Nonetheless, no molecular studies have yet confirmed a direct etiologic link between MLA and ectopic endometrium. To molecularly investigate the tumor origin, we focused on *KRAS* hotspot mutations frequently observed in MLA [6, 11, 12]. *KRAS* hotspot mutations have also been identified in non-cancerous endometriosis [13, 14], with one study reporting higher variant allele frequency accompanied by chromosomal arm-level allelic imbalances [15]. *KRAS* mutations are also detected in over one-third of adenomyosis [16]. Moreover, Inoue et al. reported that adenomyosis co-occurring with endometriosis was associated with *KRAS* mutation and low PR expression [16, 17]. As ectopic endometrium undergoes malignant transformation in certain contexts, [18] we hypothesize that *KRAS* mutations act as an early driver event in the pathogenesis of MLA, implicating *KRAS*-mutated ectopic endometrium as a precursor lesion.

In this study, to identify clinicopathological differences between MLA and EC and explore the origins of MLA, we collected resected specimens from 83 cases across multiple institutions in Japan, which were diagnosed as MLA or suspicious for MLA in the uterus or ovary. We aimed to categorize cases into MLA and non-MLA based on our morphological and immunohistochemical algorithm. Consequently, 78 and 5 of the collected cases were classified as MLAs and non-MLAs, respectively. For the 78 MLAs, we investigated the clinicopathological characteristics and origin. Whole-exome sequencing (WES) was conducted for each component in four ovarian cases (three MLAs and one non-MLA) that presented with mixed histotypes to explore the progression mechanisms of MLA.

## Methods

### Cases

Eighty-three cases (uterus, 47; ovary, 36), which were diagnosed as MLA or suspicious for MLA in 17 institutions, were included from 2008 until 2024. Three cases were previously reported as MLA (Cases 10–12) [19], along with 10 cases of OEC with MLD (Cases 28–37) [9]. Whole section hematoxylin and eosin slides of formalin-fixed paraffin-embedded (FFPE) samples were reviewed at Saitama Medical University International Medical Center (SMUIMC). Clinical information was collected from medical records at each institution.

## Diagnostic criteria of MLA

**Fig. 1:**
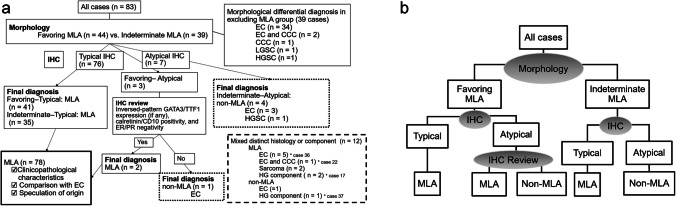
Flowchart of diagnostic criteria of mesonephric-like adenocarcinoma (MLA) and overview of the study. **a** Concise version. **b** Detailed version. All cases (*n* = 83) were morphologically categorized into favoring and indeterminate mesonephric-like adenocarcinoma (MLA) by two gynecological pathologists. For MLA-favoring cases, differential histotype, which should be ruled out, was considered. Currently, all cases are classified as typical (TTF1 and/or GATA3 ≥ 5%, ER and PR < 5%) and atypical (the other) immunohistochemistry (IHC). Cases with typical IHC were classified as MLA. Those with intermediate MLA morphology and atypical IHC were classified as non-MLA. For cases with favoring MLA morphology and atypical IHC, IHC results were reviewed, and those with any GATA3 and TTF1 expression with inverse pattern and ER and PR negativity were classified as MLA. Consequently, non-MLAs were categorized under other histotypes. Thus, 78 of the 83 cases were finally diagnosed as MLA, and clinicopathological analysis, comparison with conventional EC, and speculation of origin were conducted. Independently, mixed distinct histotype was assessed, and whole-exon sequencing was performed for each component of the four cases with an asterisk (Cases 17, 22, 36, and 37). CCC, clear cell carcinoma; EC, endometrioid carcinoma; HG, high-grade; HGSC, high-grade serous carcinoma; IHC, immunohistochemistry; LGSC, low-grade serous carcinoma; MLA, mesonephric-like adenocarcinoma

MLAs are defined as adenocarcinomas with a mesonephric-like phenotype. In this study, to distinguish MLA from other histotypes, cases were conclusively diagnosed as MLA based on specific diagnostic criteria regarding morphology and IHC (Fig. 1). Initially, cases were stratified into an MLA-favoring group and an MLA-indeterminate group based on morphology. We recorded several structural patterns with predominance (Online Resource Materials and Methods and Online Resource Fig. 1). MLA typically exhibits various histological patterns: small tubules with luminal eosinophilic colloid-like material predominate, along with an admixture of papillary-ductal, retiform, solid, or spindled architecture [5, 10]. Such morphology predominated in MLA-favoring cases. Specifically, MLA-indeterminate cases showed either MLA morphology but exhibited predominant ductal and papillary architecture, which are also characteristic of EC, or an admixture of other architecture not characteristic of MLA, such as micropapillary. Two gynecological pathologists, YM and MY, independently performed histological assessments, blinded to the IHC results. Discrepancies were resolved with discussion under a multi-head microscope, and assessments were finalized. For indeterminate MLA morphology cases, other histotypes to be excluded were noted. Subsequently, cases with TTF1 and/or GATA3 positivity and ER and PR negativity were considered typical IHC with a positive cutoff value of ≥5%.

Cases with typical IHC were classified as MLA. In addition, WT1 negativity was confirmed in the diagnosis of oMLA to exclude low-grade serous carcinoma. Those combining favoring MLA morphology with atypical IHC were classified as non-MLA. For cases with favoring MLA morphology and atypical IHC, IHC was reviewed, and those with any GATA3 and TTF1 expression with inverse patterns [6], calretinin and/or CD10 positivity, and ER and PR negativity were classified as MLA.

When a distinct histotype or component from MLA was evident, the diagnosis was “mixed MLA and distinct histotype,” detailing proportions within the carcinoma.

## Other pathological findings

Tumor diameter, gross pattern, stromal amount (predominance vs minor), pathological staging, lymphovascular space invasion (LVSI), and necrosis were morphologically assessed.

### IHC

Details on the assessment of expression for each marker are provided in the Online Resource Materials and Methods, and the primary antibodies used are listed in Online Resource Table 1.

**Table 1:** Clinicopathological characteristics of mesonephric-like adenocarcinoma (MLA) in all and each organ

	All MLA (*n* = 78)	uMLA	oMLA	*P*-value
		(*n* = 47)	(*n* = 31)	
Age (years old) (IQR)	64.5 (57–71)	65 (54–70)	63 (58–72)	0.98
Tumor diameter (cm) (IQR)	6 (4–8)	5 (4–7)	6.5 (4.5–9)	N/A
CEA (ng/mL) (IQR)	2 (1–4)	2 (1–3)	2.5 (2–6)	0.053
CA19–9 (ng/mL) (IQR)	24 (6–132)	17 (5–74)	78 (20–599)	0.02
CA125 (ng/mL) (IQR)	44 (14–136)	22 (10–60)	116 (37–475)	0.0001
Post menopause	70 (90%)	41 (87%)	29 (94%)	0.47
Pregnancy or parity history	59 (76%)	35 (74%)	24 (77%)	1
Neoadjuvant chemotherapy	3 (4%)	3 (6%)	0 (0%)	0.27
Adjuvant chemotherapy	55 (71%)	34 (72%)	21 (68%)	0.8
FIGO Stage^a^				
I	45 (58%)	23 (49%)	22 (71%)	0.1
II	10 (13%)	4 (9%)	6 (19%)	(I–II vs III–IV)
III	16 (21%)	17 (34%)	0 (0%)	
IV	6 (8%)	3 (6%)	3 (10%)	
Omental dissemination	6 (8%)	4 (8.5%)	2 (6%)	
Synchronous visceral metastasis	7 (9%)	4 (8.5%)	3 (10%)	1
Metachronous metastasis/recurrence	26 (33%)	19 (40%)	7 (23%)	0.14
Visceral metastasis^b^				
Total	21 (27%)	16 (34%)	5 (16%)	0.12
Lung	19	15	4	N/A
Liver	4	2	2	
Brain	1	1	0	
Gross pattern				
Endophytic predominant (uterus)	35 (74%)	N/A		
Solid predominant (ovary)	20 (65%)			
Differential diagnosis ^c^				
Total	35 (45%)	22 (47%)	13 (42%)	0.82
EC	31	20	11	N/A
CCC	1	0	1	
EC and CCC	2	2	0	
LGSC	1	0	1	
Mixed distinct histotypes or components				
Total	10 (13%)	1 (2%)	9 (29%)	0.0008
EC	5	0	5	N/A
EC and CCC	1	0	1	
Heterologous sarcoma (chondroid)	2	1	1	
HG component	2	0	2	
Tumor necrosis	37 (47%)	23 (49%)	14 (45%)	0.82
Minor stromal amount	61 (78%)	40 (85%)	21 (68%)	0.11
LVSI	38 (49%)	31 (65%)	7 (23%)	0.0002
Immunohistochemistry				
Thyroglobulin (%) (IQR)	0 (0–0)	0 (0–0)	0 (0–0)	1
PTEN loss	11 (14%)	8 (17%)	3 (10%)	0.2
ARID1A loss	2 (3%)	1 (2%)	1 (3%)	1
p53 mutant pattern	0 (0%)	N/A		
Deficient mismatch repair	0 (0%)	N/A		
Tumor *KRAS* exon 2 codon 12 mutation	66 (85%)	36 (77%)	30 (97%)	0.022

*CCC* clear cell carcinoma, *CEA* carcinoembryonic antigen, *CA* carbohydrate antigen, *EC* endometrioid carcinoma, *HG* high-grade, *HGSC *high-grade serous carcinoma, *IQR* interquartile range, *LGSC* low-grade serous carcinoma, *LVSI* lymphovascular space invasion, *MLA* mesonephric-like adenocarcinoma, *N/A* not assessed, *oMLA* ovarian mesonephric-like adenocarcinoma, *uMLA* uterine mesonephric-like adenocarcinoma

^a^2009 staging system for the uterus and the 2014 staging system for the ovary

^b^Both synchronous and metachronous metastases were included, with overlapping organs, such as the lung and liver, being counted

^c^Assessed in morphologically indeterminate MLA cases

## Morphological speculation of origin

### Uterus

We morphologically speculated the origin of the tumor based on the adjacent preexisting lesion and tumor location (Online Resource Fig. 2). We identified preexisting lesions from which carcinoma could develop, including endometrioid intraepithelial neoplasia (EIN), endometrial polyp (EP), and adenomyosis. Among these, only the lesions adjacent to the tumor were considered. Tumor involvement in the endometrium and/or myometrium was assessed based on predominance. Finally, the morphologically speculated origin was determined.

## Ovary

Possible origins of the tumor included the following epithelial structures: endometriosis, mesonephric remnant, Walthard nest, and parafallopian cyst. Among them, only sites of endometriosis were found adjacent to MLA, which was considered a morphologically speculated origin.

## KRAS mutation analysis

For MLA, other coexisting histotypes, and morphologically speculated origins, *KRAS* codon 12 hotspot mutation analysis was conducted. As the suspected origins generally comprised only a minor portion of the sample, laser-capture microdissection was performed using an LMD 7000 instrument (Leica Microsystems, Wetzlar, Germany) on the 10 µm-thick FFPE samples to extract DNA. The estimated targeted cell ratio was 60–90%. The procedure of DNA extraction and *KRAS* hotspot mutation analysis using Sanger sequencing is described in the Online Resource Materials and Methods. The primers used for sequencing are listed in Online Resource Table 2.

### WES

Four ovarian cases coexisting with other histotypes were selected. The procedures of DNA extraction, WES, and data analysis are described in the Online Resource Materials and Methods.

## Control cases

We utilized clinicopathological data from 100 endometrial endometrioid carcinomas (EECs) and 100 OECs from SMUIMC, along with 232 ECs from The Cancer Genome Atlas [20]. Detailed information is described in the Online Resource Materials and Methods.

## Statistics

Progression-free survival (PFS) and overall survival (OS) were estimated using the Kaplan–Meier method. Survival analyses were conducted separately for uMLA and oMLA. For each organ, univariate Cox proportional hazards models were fitted. Variables with *P* < 0.20 in univariate analysis were included in multivariate models. Statistical significance was set at *P* < 0.05. FIGO stage comparisons (I vs II, I vs III, and I vs IV) were modelled independently to avoid instability. All analyses were conducted using Python (lifelines 0.30.0) and JMP Pro 17 (SAS Institute Inc., Cary, NC, USA).

## Ethics

All participants provided informed consent. The ethics review board of SMUIMC approved the study design as a centralized review (approval number: 2022-067).

## Results

### Case selection

**Fig. 2:**
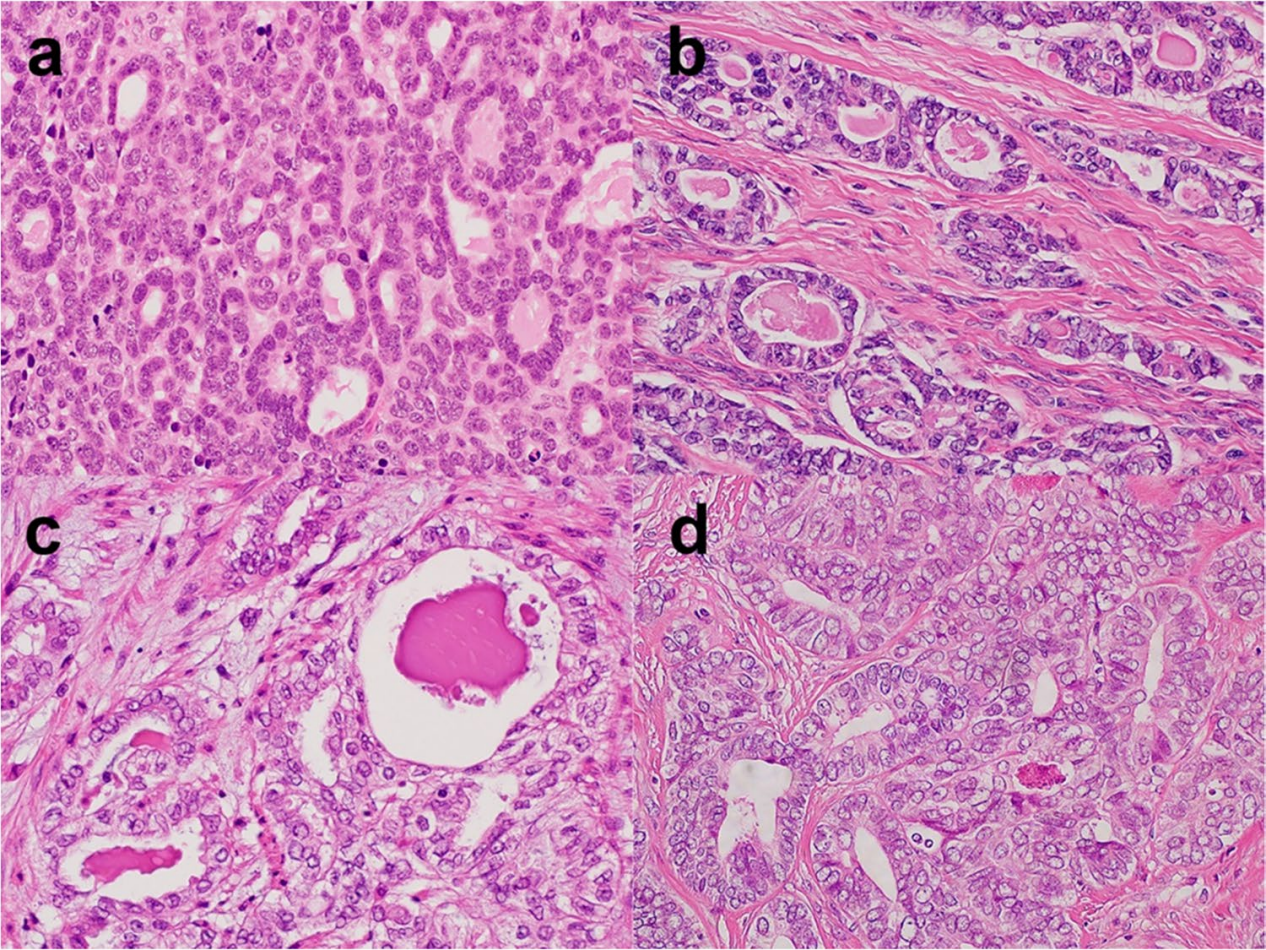
Representative microphotographs of mesonephric-like adenocarcinoma (MLA). **a** Uterine MLA (uMLA), favoring MLA morphology (Case 71). High nuclear to cytoplasmic (N/C) ratio cells formed tubular architecture. Colloid-like secretions were observed in the lumen. **b** Ovarian MLA (oMLA), favoring MLA morphology (Case 13). High N/C ratio cells formed the tubular architecture. **c** uMLA, indeterminate MLA morphology (Case 47). N/C ratio was low; however, colloid-like secretions were found in the tubular lumen. **d** oMLA, indeterminate MLA morphology (Case 31). Intermediate high-columnar cells formed tubular and ductal architecture. Colloid-like secretions were slightly observed

All 83 cases were classified into 78 MLA and 5 non-MLA cases based on the diagnostic criteria shown in Fig. 1. Representative micrographs of MLA and non-MLA are shown in Fig. 2 and Online Resource Fig. 3, respectively. One non-MLA, an OEC, had a high-grade (HG) component and was previously reported as an OEC with MLD coexisting with dedifferentiated carcinoma [9]. Online Resource Table 3 compares pathological differences between MLA and non-MLA. Full clinicopathological data are included in Online Resource Table 4.

## Clinicopathological characteristics and prognostic factors

Table 1 presents the clinicopathological characteristics of MLAs. Nine of the 31 (29%) oMLAs harbored mixed distinct histotypes or components with a variable range (10–95%). However, only one uMLA coexisted with heterologous sarcoma.

Among 47 patients with uMLA, PFS was 1 to 92 months (median = 15 months) and OS was 1 to 127 months (median = 25 months). During the follow-up period, 20 patients (42.6%) progressed and 11 (23.4%) died. Among 31 patients with oMLA, PFS was 1 to 107 months (median = 21 months) and OS was 1 to 107 months (median = 26 months). During the follow-up period, seven patients (22.6%) progressed and four (12.9%) died. Among 100 patients with EEC, PFS was 1 to 75 months (median = 62 months) and OS was 4 to 76 months (median = 62.5 months). During the follow-up period, seven patients (7.0%) progressed and five (5.0%) died. Among 100 patients with OEC, PFS was 0 to 87 months (median = 35.5 months) and OS was 0 to 97 months (median = 57). During the follow-up period, 14 patients (14.0%) progressed and 7 (7.0%) died.

Prognostic factors of progression are summarized in Table 2. In uMLA, there were 20 progression events among 47 patients. LVSI was an independent and significant poor prognostic factor in multivariate analysis (*P* = 0.003 [HR: 11.33, 95% CI: 1.26–101.69]). However, factors other than LVSI did not reach significance in the multivariate analysis, and the wide confidence intervals warrant cautious interpretation. In oMLA, there were only 7 progression events among 31 patients. Accordingly, multivariate analysis using the Cox proportional hazards model was not performed.

**Table 2:** Prognostic factors of progression in mesonephric-like adenocarcinoma (MLA)

	uMLA (*n* = 47)				oMLA (*n* = 31)	
	Univariate HR (95% CI)	*P*-value	Multivariate HR (95%CI) ^a^	*P*-value	Univariate HR (95% CI)	*P*-value
Morphology (MLA-favoring vs MLA-indeterminate)	0.88 (0.37–2.13)	0.78	—	—	0.50 (0.11–2.24)	0.78
Predominant growth (endophytic vs exophytic/solid)	3.10 (0.90–10.65)	0.072	1.15 (0.30–4.38)	0.84	N/A^b^	N/A
Tumor necrosis (Present vs Absent)	2.46 (0.98–6.21)	0.056	1.94 (0.71–5.29)	0.19	N/A^b^	N/A
LVSI (Present vs Absent)	13.45 (1.78–101.86)	0.012	11.33 (1.26–101.69)	0.03	3.03 (0.68–13.64	0.15
Stage I (23) vs II (4)	1.81 (0.21–15.78)	0.59	—	—	N/A^c^	N/A
Stage I (23) vs III (17)	0.20 (0.07–0.6)	0.0039	—	—	N/A^c^	N/A
Stage I (23) vs IV (3)	0.30 (0.07–1.27)	0.1	—	—	N/A^c^	N/A

*CI* confidence interval, *HR* hazard ratio, *LVSI* lymphovascular space invasion, *MLA* mesonephric-like adenocarcinoma, *N/A* not assessed, *oMLA* ovarian mesonephric-like adenocarcinoma, *uMLA* uterine mesonephric-like adenocarcinoma

The International Federation of Gynecology and Obstetrics (FIGO) 2009 staging system for the uterus and the 2014 staging system for the ovary

^a^For uMLA, the multivariate analysis included factors with *P* < 0.20 in the univariate analysis (LVSI, tumor necrosis, predominant growth pattern)

^b^In oMLA, no progression events occurred in the tumor necrosis–negative group or in the solid/cystic group; therefore, hazard ratios could not be estimated

^c^In oMLA, the numbers of FIGO stage II (*n* = 6), III (*n* = 0), and IV (*n* = 3) cases were extremely small and the Cox model failed to converge; estimates were, thus, not obtainable

## Clinicopathological comparison between uMLA and EEC

**Fig. 3:**
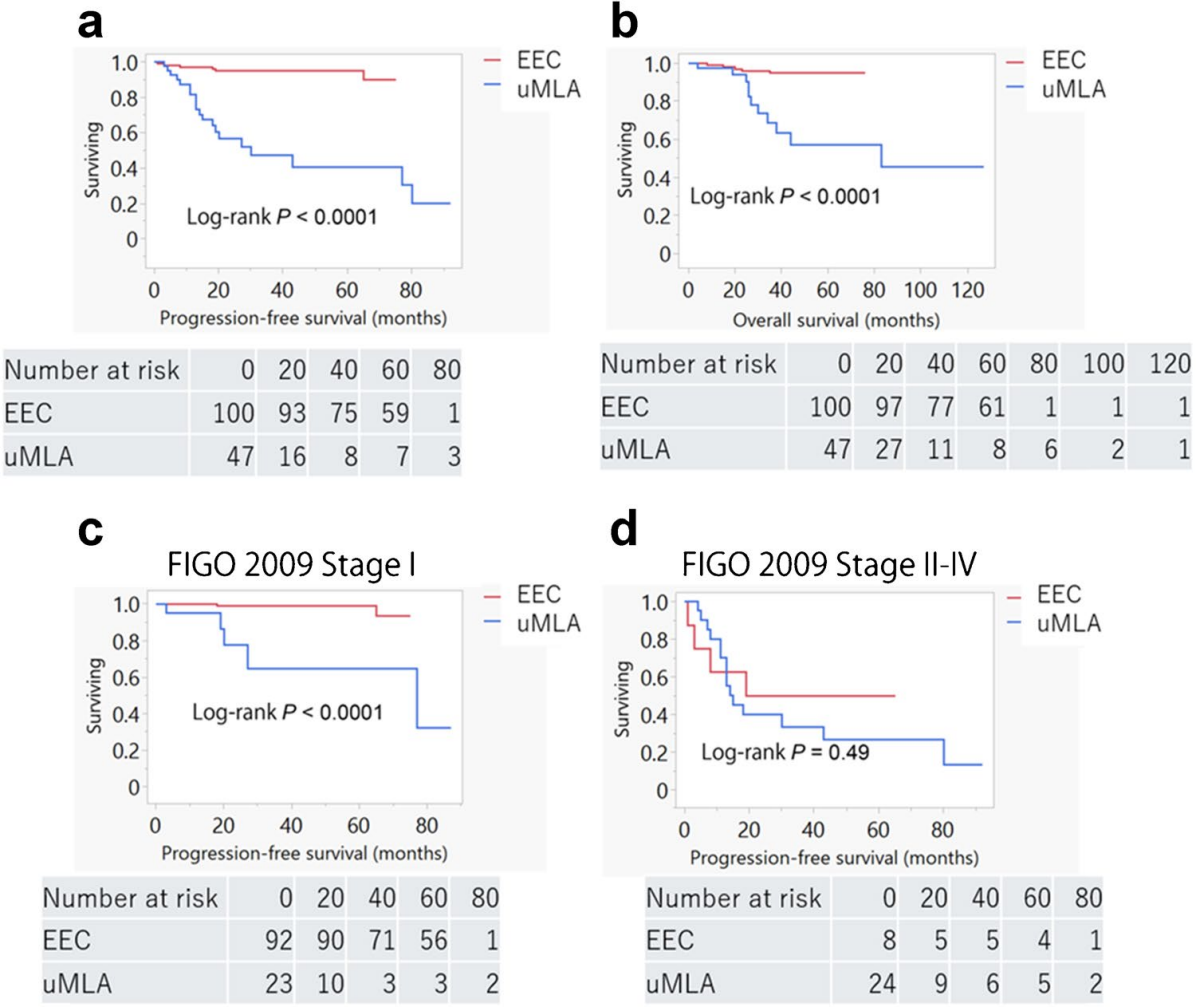
Survival curve of uterine mesonephric-like adenocarcinoma (uMLA) and endometrial endometrioid carcinoma (EEC). **a** Progression-free survival (PFS) of uMLA was worse than that of EEC. **b** Overall survival of uMLA was worse than that of EEC. **c** PFS of uMLA was worse than that of EEC in the International Federation of Gynecology and Obstetrics (FIGO) stage I. **d** PFS was not different between uMLA and EEC in FIGO stage II–IV

uMLA occurred in older patients (*P* = 0.01), presented at a more advanced stage (*P* < 0.0001), was associated with more frequent LVSI (66%, *P* < 0.0001), and had a greater presence of adenomyosis (62%, *P* = 0.0005) than EEC (Table 3). The PFS and OS for uMLA were worse than those for EEC (Fig. 3a, b). The OS did not vary between uMLA and EEC when divided into stage I and II–IV (Online Resource Fig. 4a, b). Although the PFS for uMLA was inferior to that for EEC in stage I or I–II, no differences were observed in stage II–IV or III–IV (Fig. 3c, d and Online Resource Fig. 4c, d). The PFS and OS of uMLA were comparable to those of copy number-high tumors in integrated subtypes of EC (Online Resource Fig. 5).

**Table 3:** Comparison of uterine mesonephric-like adenocarcinoma (uMLA) and endometrioid endometrial carcinoma (EEC)

	uMLA	EEC	*P*-value
	(*n* = 47)	(*n* = 100)	
Age (years old) (IQR)	65 (55–70)	59 (50–68)	0.01
FIGO Stage 2009			
I	23 (49%)	92 (92%)	<0.0001 (I vs II–IV)
II	4 (8.5%)	2 (2%)	
III	17 (36%)	6 (6%)	
IV	3 (6.5%)	0 (0%)	
FIGO I			
a	10 (43%)	76 (83%)	0.0003
b	13 (57%)	16 (17%)	
Endophytic growth	35 (74.5%)	14 (14%)	<0.0001
Tumor location			
E > M	13 (28%)	86 (86%)	<0.0001
Limited in M	6 (13%)	0 (0%)	
M > E	28 (59%)	14 (14%)	
Histological grade			
1	N/A	72 (72%)	N/A
2		16 (16%)	
3		12 (12%)	
LVSI	31 (66%)	23 (23%)	<0.0001
EIN	4 (8.5%)	74 (74%)	<0.0001
Adenomyosis			
All adenomyosis	29 (62%)	28 (28%)	0.0005
Adjacent adenomyosis	23 (49%)	7 (7%)	<0.0001

*E* endometrium, *EIN* endometrioid intraepithelial neoplasia, *EEC* endometrioid endometrial carcinoma, *FIGO* the International Federation of Gynecology and Obstetrics, *IQR* interquartile range, *LVSI* lymphovascular space invasion, *M* myometrium, *uMLA* uterine mesonephric-like adenocarcinoma, *N/A* not assessed

## Clinicopathological comparison of oMLA and OEC

**Fig. 4:**
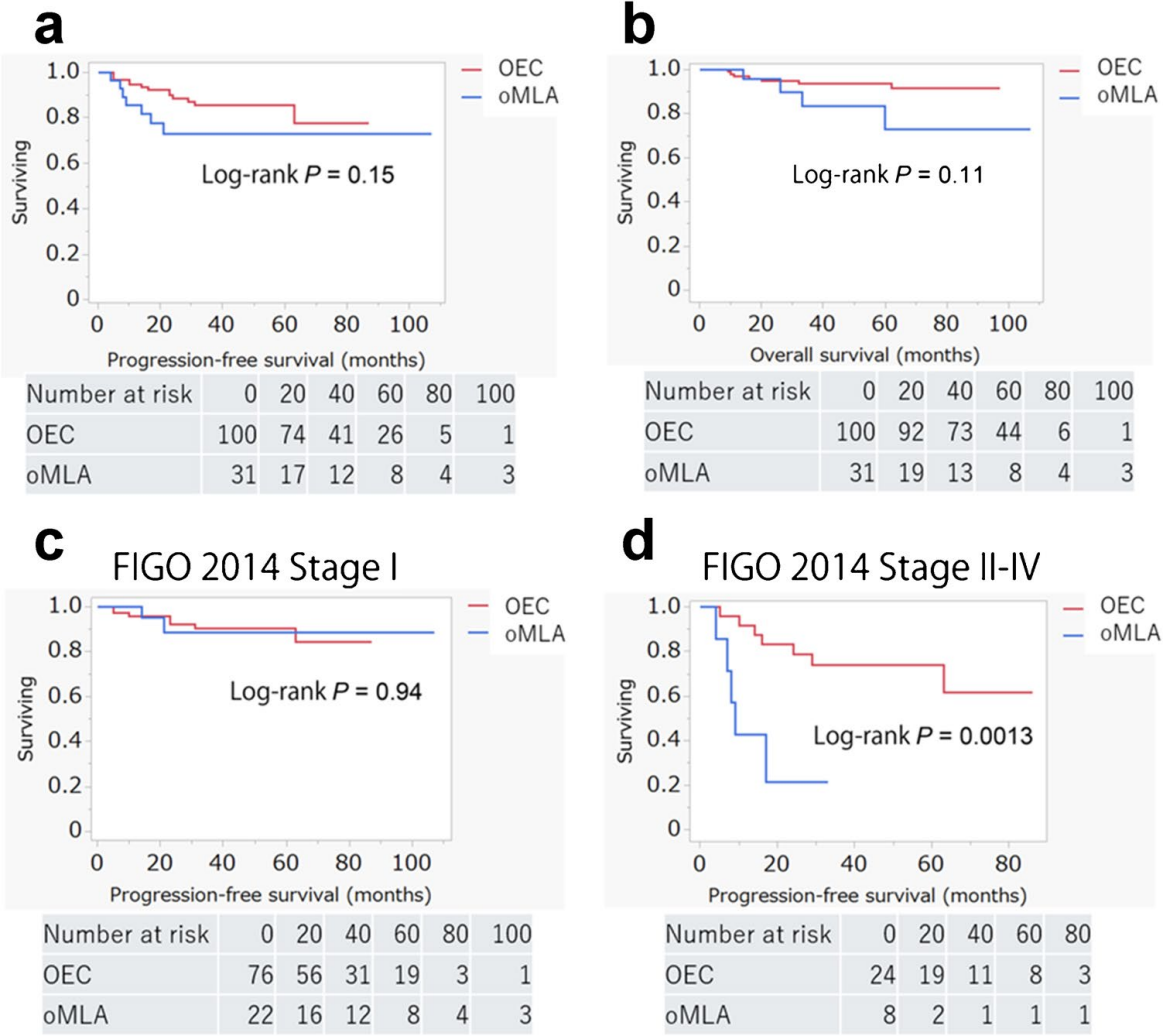
Survival curve of ovarian mesonephric-like adenocarcinoma (oMLA) and ovarian endometrioid carcinoma (OEC). **a** Progression-free survival (PFS) was not different between oMLA and OEC. **b** Overall survival was not different between oMLA and OEC. **c** PFS was not different between oMLA and OEC in the International Federation of Gynecology and Obstetrics (FIGO) stage I. **d** PFS of oMLA was worse than that of OEC in FIGO stage II–IV

FIGO stage, gross pattern, and LVSI were similar between oMLA and OEC (Online Resource Table 5). oMLA had a worse PFS and OS than OEC in stage II–IV, but not across all stage I, early (I–II), or advanced (III–IV) stages (Fig. 4 and Online Resource Fig. 6). This tendency was not different in the 22 pure oMLAs without other histotypes (Online Resource Fig. 7).

## Origin of MLA

In uMLAs, the origin was speculated using an algorithm illustrated in Online Resource Fig. 2, with two representative cases shown in Online Resource Fig. 8. Table 4 summarizes the results of *KRAS* hotspot mutation analysis of uMLA. Among the 36 uMLAs that harbored a *KRAS* mutation, endometriosis specimens shared the same mutation with the tumor most frequently (12 [33%]), followed by the endometrium (4 [11%]) and EIN (3 [8%]).

**Table 4:** *KRAS* hotspot mutation of uterine mesonephric-like adenocarcinoma (uMLA) and morphologically speculated origins

		*KRAS* hotspot mutation		
	All	Present	Absent	N/A
uMLA	47	36	11	0
Morphologically speculated origin				
E	34 (72%)	4^a^	17	13^b^
Adenomyosis	21 (45%)	12^a^	6	3^c^
EIN	4 (8.5%)	3	1	0
EP	3 (6%)	0	3	0

Underline indicates a molecularly speculated origin

*E* endometrium, *EIN* endometrioid intraepithelial neoplasia, *EP* endometrial polyp, *uMLA* uterine mesonephric-like adenocarcinoma, *N/A* not assessed

^a^One case harbored *a KRAS* mutation in both E and adenomyosis

^b^Reason for N/A: six cases were wild-type tumors, and seven cases were shortage of DNA

^c^Reason for N/A: two cases were wild-type tumors, and one case was a shortage of DNA

In total, 29 of the 31 (94%) oMLAs harbored endometriosis (Table 5), and 23 of these endometrioses (79%) shared the same *KRAS* mutation with the tumor.

**Table 5:** *KRAS* hotspot mutation of ovarian mesonephric-like adenocarcinoma (oMLA), possible origin, coexisting borderline tumor, and mixed distinct histotype

		*KRAS* hotspot mutation			
	All	Present	Present but different from that of the oMLA	Absent	N/A
oMLA	31	30	N/A	1	0
Possible origin					
Endometriosis	29 (94%)	23	1^d^	5	0
Mesonephric remnant	5 (16%)	1^a^	0	1	3
Paratubal cyst	1 (3%)	1^a^	0	0	0
Walthard nest	1 (3%)	0	0	1	0
Coexisting borderline tumor					
Endometrioid^b^	6 (19%)	2	1^d^	0	3
Seromucinous^b^	1 (3%)	1	0	0	0
Mucinous	1 (3%)	1	0	0	0
Mixed distinct histotype/component					
EC	5 (16%)	3	0	0	2
Sarcoma	1 (3%)	0	0	0	1
HG component	2 (6%)	2	0	0	0
EC and CCC	1 (3%)	1^c^	0	0	0

Among possible origins, only endometriosis was found adjacent to MLA and, thus, was regarded as a morphologically speculated origin. Underline indicates a molecularly speculated origin

*CCC* clear cell carcinoma, *EC* endometrioid carcinoma, *HG* high-grade, *MLA* mesonephric-like adenocarcinoma, *N/A* not assessed

^a^Endometriosis harbored a common *KRAS* mutation with MLA was present

^b^Endometriosis coexisted in the background

^c^Each component possessed a *KRAS* mutation (Case 22)

^d^Same case (Case 12)

## Genetic differences between oMLA and other histotypes

We conducted WES for four ovarian cases with distinct histotypes (Cases 17, 22, 36) and non-MLA (Case 37). The detailed results are shown in Online Resource Results, Online Resource Figs. 9–15, and Online Resource Tables 6–11.

## Discussion

**Fig. 5:**
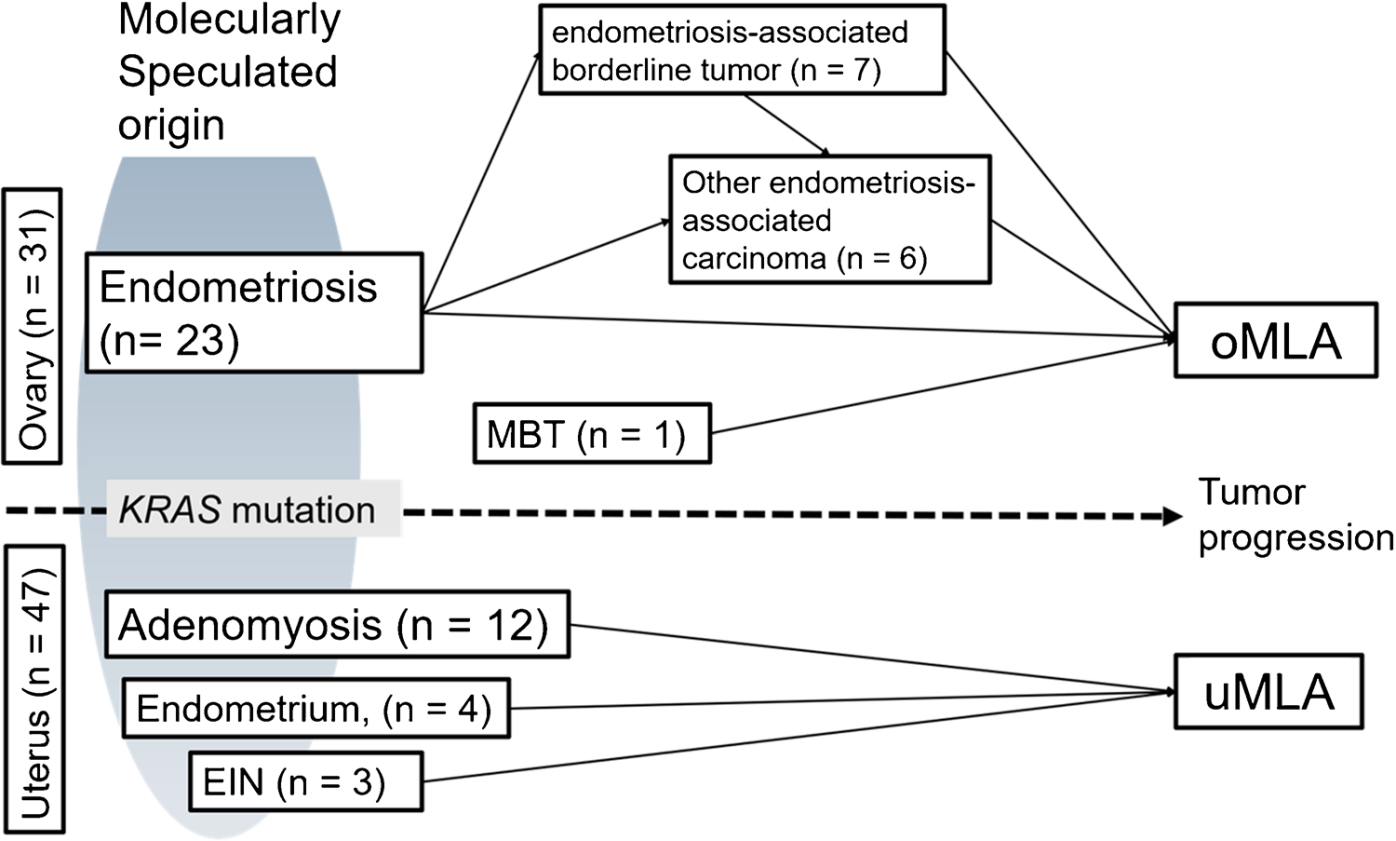
Putative tumor progression of ovarian and uterine mesonephric-like adenocarcinoma (MLA). In the ovary, as endometriosis and mucinous borderline tumor (MBT) harbored common *KRAS* mutations with ovarian MLA (oMLA), they were considered speculated origin. Several cases develop into other endometriosis-associated carcinomas, such as endometrioid carcinoma or endometriosis-associated borderline tumor. In the uterus, adenomyosis, followed by endometrium and endometrioid intraepithelial neoplasm (EIN), harbored a common *KRAS* mutation with uterine MLA (uMLA)

In this study, we aimed to elucidate the clinicopathological characteristics of MLA. Moreover, to explore the origins of MLA, we reviewed the whole section slides of each case and analyzed *KRAS* hotspot mutations in the morphologically speculated origins and MLA. To the best of our knowledge, this is the first study to molecularly substantiate the link between MLA and ectopic endometrium. The putative tumor progression of MLA is depicted in Fig. 5.

We set the diagnostic criteria for MLA based on morphological and immunohistochemical evaluations. It is crucial to morphologically suspect the MLA and conduct IHC. Specifically, MLA frequently shows GATA3, TTF1, calretinin, and CD10 positivity, and ER negativity [1, 3, 7, 8, 21, 22]. Among them, several researchers have suggested that hormone receptor negativity and a combination of TTF1 and GATA3 expression are useful diagnostic markers [3, 8]. Although the cutoff value of positivity is controversial, we set the positive cutoff at ≥5% for initial assessment. However, for cases with favoring MLA morphology, ER and PR negativity, and an inverse pattern of TTF1 and GATA3, even if they were expressed in <5%, were rendered significant because Euscher et al. reported this pattern to be characteristic of MLA [6]. We also confirmed calretinin and CD10 expression. Consequently, three cases with favoring MLA morphology and atypical IHC were classified as MLA.

Köbel et al. suggested a low threshold for morphological features of MLA to recommend an ancillary immunohistochemical marker panel, including PAX2, hormone receptor, GATA3, and TTF1 [8]. We concur with their proposal and categorized cases into typical and atypical IHC. Nevertheless, several cases exhibited both ER and GATA3 and/or TTF1 expression. Köbel et al. reported cases with both OEC morphology and TTF1 and/or GATA3 expression (≥1%) and varying levels of ER and/or PR expression, showing a prognosis similar to that of MLA [8]. However, we recommend cautious interpretation of cases exhibiting ER and/or PR expression, which aligns with prior studies indicating that “patchy” or low-level ER expression can be consistent with MLA [23, 24].

Among the five non-MLA, four were diagnosed as OEC, and the remaining was identified as high-grade serous carcinoma (HGSC), exhibiting TTF1, GATA3, and ER positivity. Although this case exhibited indeterminate MLA morphology, severe nuclear atypia and marked mitotic figures were observed (Online Resource Fig. 2d). p53 exhibited a null pattern, indicating aberrant *TP53* status. Euscher et al. and Mills et al. reported two similar cases, harboring p53 abnormality and *KRAS* hotspot mutation [3, 24]. Although whether they should be classified as HGSC or oMLA is debatable, we categorized them as HGSC and emphasized that HGSC could also harbor a mesonephric-like phenotype.

Nine of the 31 (30%) oMLAs and one uMLA presented mixed distinct histotypes. The most frequent mixed histotype was EC, which exhibited ER positivity. One uMLA had chondroid elements, which appear to be a characteristic of mesonephric-like carcinosarcoma [25]. Although not included in this study, Yano et al. reported a case of mixed uMLA and EEC, where hormone therapy was effective only for EEC [26]. Our study showed no instances of coexisting serous tumors, germ cell tumors, or sex cord-stromal tumors previously reported in other studies [3, 27–31].

uMLA presented a more advanced stage and a poorer prognosis than conventional EEC, consistent with a retrospective review by Kim et al., which analyzed the seven uMLAs extracted from 237 ECs [32]. In our study, 23/47 (49%) cases of uMLAs were in stage II–IV, showing a slightly lower frequency than that previously reported by Pors et al. and Kim et al. (18/25 and 25/43, respectively) [2, 33]. Furthermore, the median PFS of the uMLAs, 30 months, was longer than that reported by Pors et al. and Euscher et al. (18–21 months) [2, 6]. Nevertheless, it is noteworthy that uMLA had worse PFS than EEC, even when limited to stage I (Fig. 3c) or I–II (Online Resource Fig. 8c). This might be explained by the fact that stage I uMLA had more frequent deep myometrial invasion (uMLA 57% vs EEC 17% [*P* = 0.003], Table 3) or that LVSI was an independent and significant poor prognostic factor of uMLA in multivariate analysis (*P* = 0.003 [HR: 11.33, 95% CI: 1.26–101.69].

In the oMLA of our study, the prognosis of stage I was favorable, similar to that of OEC. However, Köbel et al. compared disease-specific survival between 14 oMLAs and 157 OECs limited to stage I and found a considerably worse prognosis for oMLA than for OEC [8]. This discrepancy between their results and ours may be due to different MLA selection criteria, such as IHC panels or expression cutoff values. However, the small number of stage I oMLAs makes it difficult to conclude their prognosis.

MLA is currently considered to be of Mullerian origin [10]. oMLAs are commonly associated with endometriosis [1, 34–36]. Several studies have revealed that mutation and loss of expression of *ARID1A* are observed in the endometriosis immediately adjacent to EC or CCC in the ovary [37, 38]. Similar to these endometriosis-associated carcinomas, we hypothesized that *KRAS* hotspot mutations, frequently observed in oMLA [6, 11, 12], initially occur in the tumor progression of oMLA before being recognized as a tumor. Twenty-three of the 29 (79%) oMLAs with *KRAS* mutations harbored a common one associated with endometriosis. One case of oMLA coexisting with a mucinous borderline tumor harbored a common *KRAS* G12C mutation. Although Nilforoushan et al. identified four cases of ovarian mucinous tumors coexisting with mesonephric-like lesions sharing common *KRAS* hotspot mutations [39], Sim et al. reported that in the ovarian mucinous tumor populations, mesonephric-like components were rarely detected [40]. Additionally, Mezzapesa et al. reported five oMLAs that were all categorized as endometriosis-correlated carcinoma, occupying 10% of this category [41].

Three EINs and four endometria were identified as the molecularly speculated origin of uMLA. Santoro et al. reported mesonephric-like lesions in the endometrium [42], whereas Lac et al. found that the *KRAS* codon 12 hotspot mutation appears in 28% (31/110) of normal endometrium from women lacking evidence of gynecologic malignancy or EIN [43]. Therefore, we propose that the endometrium with *KRAS* hotspot mutations may evolve into uMLA. Conversely, adenomyosis was noted in uMLA two times as often as in EEC (62% vs 28%). Yamamoto et al. also reported magnetic resonance imaging findings indicating that coexisting adenomyosis is present in over 50% of patients with uMLA [44]. In our molecular analysis, among 36 uMLAs harboring *KRAS* hotspot mutations in tumors, 12 (33%) had common mutations with adenomyosis, which also indicated 12/21 (67%) of adenomyosis had *KRAS* mutations. Additionally, six uMLAs were confined to the myometrium (13%). Cases confined to the myometrium without endometrial involvement might be analogous to those reported as uterine mesonephric adenocarcinoma without mesonephric remnant [45–47], with adenomyosis in one case [48]. Among previously reported cases of EC arising in adenomyosis, we found one case that morphologically seemed to resemble uMLA [49]. Moreover, in a systematic review of ER expression in ECs arising in adenomyosis by Machida et al., 12 of the 14 (86%) cases were ER-negative [50].

This study had some limitations. First, the number of cases and events in the oMLA cohort was small. In oMLA, progression events were limited to 7 of 31 cases overall, and the stage II–IV subgroup was even smaller. Although the uMLA cohort had a larger number of progression events (*n* = 20), factors other than LVSI did not emerge as independent prognostic variables in multivariate analysis, and the confidence intervals were wide. Thus, validation in larger cohorts is warranted. Second, we molecularly speculated the origin of MLA based on the presence of a common *KRAS* codon 12 hotspot mutation between MLA and an adjacent lesion through Sanger sequencing. However, analyses on some morphologically speculated origins were not performed owing to insufficient DNA quantity. Third, the other mutations reported to be observed in MLA, including *PIK3CA*, *NRAS*, and *BRAF*, were not analyzed [6, 11].

In conclusion, we revealed the clinicopathological characteristics of MLAs. Especially, among 29 oMLAs harboring a *KRAS* hotspot mutation, 23 (79%) instances of endometriosis in the background had the same mutation. Moreover, among 36 uMLAs carrying the *KRAS* hotspot mutation, common mutations were observed in 12 (33%) instances of adjacent adenomyosis. Our hypothesis regarding the close histogenetic association of MLA with ectopic endometrium harboring the *KRAS* mutation can be anticipated to contribute immensely to the pathological and clinical analysis of MLA.

## Supplementary Information

Below is the link to the electronic supplementary material.

Supplementary file 1 (DOCX 18.0 MB)

Supplementary file 2 (XLSX 150 KB)

Supplementary file 3 (XLSX 1.12 MB)

Supplementary file 4 (XLSX 96.2 KB)

## Data Availability

All data generated or analysed during this study are included in Online Resource Table 4.
