# Supplementary material for: Multi-institutional study of mesonephric-like adenocarcinoma: its clinicopathological characteristics and histogenetic association with ectopic endometrium
Source: Virchows Arch. 2025 Oct 29;489(2):441–54. doi: 10.1007/s00428-025-04286-0 (PMC13477489; doi:10.1007/s00428-025-04286-0)
Supplement: Supplementary file 1 [file 428_2025_4286_MOESM1_ESM.docx]

**Supplementary material**

Multi-institutional study of mesonephric-like adenocarcinoma: Its clinicopathological characteristics and histogenetic association with ectopic endometrium

Virchows Archiv

Yu Miyama, Kousuke Uranishi, Masataka Hirasaki, Akiko Tonooka, Fumi Kawakami, Yoshiki Mikami, Takako Kiyokawa, Hiroshi Kajiwara, Junichi Shiraishi, Eiji Kudo, Kozue Ito, Morihiro Higashi, Sachiko Minamiguchi, Nasa Okazaki, Yuzuru Kondo, Tetsuya Shimada, Yoshinobu Maeda, Yumiko Yasuhara, Yoshiaki Kawano, Yohei Kawasaki, Kosei Hasegawa, Masanori Yasuda ^*^

***Correspondence:**

Masanori Yasuda

# Department of Pathology, Saitama Medical University International Medical Center, Yamane 1397-1, Hidaka, Saitama, Japan, 350-1298, Tel: +81-42-984-6090; Fax: +81-42-984-6090; E-mail: [m_yasuda@saitama-med.ac.jp](about:blank)

**Materials and Methods**

**Structural patterns**

We recognised several architectures (Online Resource Figure 1), including small tubules, spindled, and glomeruloids, which are characteristic of mesonephric-like adenocarcinoma (MLA). Additionally, ductal and papillary architectures, overlapping between MLA and endometrioid carcinoma (EnC), and solid architectures, overlapping among MLA, high-grade serous carcinoma (HGSC), and EnC, were identified. Architectural patterns characteristic of other histotypes not associated with MLA were also evaluated, including tubulocystic architecture typical of clear cell carcinoma (CCC) and labyrinthine or micropapillary architecture typical of HGSC. The most predominant architecture was noted. In cytology, predominant nuclear chromatin (fine or coarse) and the nuclear to cytoplasmic ratio (N/C), using a 0.8 cutoff, were assessed. The presence of hobnail-like cells and squamous metaplasia was documented, regardless of quantity. Additionally, tumour cells with abundant clear cytoplasm, typically forming nests and frequently observed in MLA, were recorded.

# Immunohistochemistry (IHC)

Formalin-fixed paraffin-embedded (FFPE) sections of 4 μm thickness were used. The primary antibodies employed are listed in Online Resource Table 1. Visualisation was achieved using an iView DAB Universal Kit (Roche Diagnostics, Basel, Switzerland), with haematoxylin serving as a counterstain. For TTF1, ER, PgR, CD10, GATA3, calretinin, thyroglobulin, and WT1, the proportion of expression was recorded. Luminal expression of CD10, nuclear and cytoplasmic expression of calretinin, cytoplasmic expression of thyroglobulin, and nuclear expression of the other markers were recorded. WT1 was investigated only for oMLA to exclude low- and high-grade serous carcinoma. When other histotypes coexisted, assessment was confined to the component with confirmed or suspected MLA morphology.

For PTEN and ARID1A, absent expression was considered a loss of function. [1, 2] Moreover, diffuse or absent p53 expression was evaluated as aberrant (mutant type), as previously reported. [3]

## **Mismatch repair (MMR) status**

Microsatellite instability (MSI) testing or MMR IHC was performed. MSI testing with FFPE samples utilised the pentaplex reaction method. [4] MMR IHC employed a modified approach using only MSH6 and PMS2. [5] The primary antibodies for MSH6 (clone SP93) and PMS2 (clone A16-4) were used, with visualisation achieved through an OptiView DAB Universal Kit (Roche Diagnostics).

## **DNA extraction and *KRAS* hotspot mutation analysis using Sanger sequencing**

DNA samples were extracted using a Maxwell DNA FFPE Kit (Promega, Madison, WI, USA). The extracted DNA samples were amplified via polymerase chain reaction (PCR) using three pairs of primers specific to the *KRAS* gene with the codon 12 hotspot mutation (Online Resource Table 2). The PCR products were purified using ExoSAP-IT (Applied Biosystems, Waltham, MA, USA) and a QIAquick Gel Extraction Kit (QIAGEN, Hilden, Germany). Subsequently, the isolated PCR products were subjected to Sanger sequencing using the BigDye Terminator version 3.1 cycle sequencing kit (Applied Biosystems) and analysed on 3500 Genetic and 3730xl DNA analysers (Applied Biosystems). The sequence chromatogram files were examined using Chromas version 2.6.6 (Technelysium, South Brisbane, Australia), MEGA X version 10.1.8 (Pennsylvania State University, University Park, PA, USA), and SeqMan Pro version 10 (DNASTAR, Madison, WI, USA).

### **Whole-exome sequencing (WES) and data analysis**

We conducted WES for the following two mixed ovarian MLAs (oMLAs) with distinct histotypes: endometrioid tumour (benign-borderline), oMLA, ovarian endometrioid carcinoma (OEC), and CCC (Case 22); and oMLA and OEC (Case 36). Additionally, analyses were performed on oMLA (Case 17) and non-MLA: OEC with MLD (Case 37), both containing high-grade (HG) components.

Exome enrichment utilised the Twist Exome 2.0 Kit (Twist Bioscience HQ, South San Francisco, CA, USA), and the prepared DNA libraries were sequenced using NovaSeq 6000 (Illumina, San Diego, CA, USA) with paired-end 151 bp reads at Azenta Ltd. (Tokyo, Japan). The identification of somatic variants relied on previously reported methods, with some adjustments. Using uterine or omental tissue sample data as a normal reference, Mutect2 from the Genome Analysis Toolkit (GATK) was employed to identify somatic cell-derived single-nucleotide variants (SNVs) and small INDELs. [6] Variants detected via Mutect2 were filtered using GATK FilterMutectCalls, and those determined to be “Pass” were annotated with ANNOVAR. [7] All SNV data are presented in Online Resource Table 6.

The next-generation sequencing data analysis only reported the presence of AltSeq (Alt, any other allele found at that locus); therefore, if no sequence reads were observed, AltSeq was considered absent. We extracted pathogenic variants from column “CLNSIG” labeled with “Pathogenic,” “Likely pathogenic,” and “Pathogenic/Likely pathogenic.” For cases with HG components, frameshift insertion/deletion or stopgain variants associated with the SWItch/Sucrose Non-Fermentable complex were also detected.

Variant filtering based on criteria such as depth of coverage, variant allele frequency (VAF), and AltSeq counts reduces false-positive results and ensures confidence in the detected variants. VAF was calculated by dividing AltSeq counts by the sum of AltSeq and RefSeq counts. By setting thresholds for “Depth of coverage ≥15, VAF ≥20%, and AltSeq ≥ 2,” we aimed to ensure that detected variants were supported by sufficient sequencing reads and were present at a significant level to be considered genuine. These exonic functional variants are summarised in Online Resource Tables 7–10.

Radar analysis was used to detect copy number variations (CNVs), and the pipeline was based on previously reported methods, [8] using uterine or omental tissue sample data as a normal reference. The HaplotypeCaller in GATK was used to perform local re-alignment of the reads around the indel, and single-nucleotide polymorphisms in the genomes of normal and tumour samples were identified. ^[6]^ By setting thresholds for “Observed Depth ≥70” and “Expected Depth ≥70,” we aimed to ensure that sufficient sequencing supported detected variant reads and were present at a significant level to be considered genuine. All CNV Radar data are shown in Online Resource Table 11 and Online Resource Figures 10–13.

### **Control cases**

### **Endometrial** **endometrioid carcinoma (EEC) and OEC**

Patients who underwent hysterectomy for endometrial carcinoma (EC) between January 2018 and February 2019, as well as those who underwent salpingo-oophorectomy for ovarian carcinoma from March 2014 to May 2021 at Saitama Medical University International Medical Center, were enrolled. Upon review by two gynaecological pathologists, 100 serial cases each of conventional EEC and OEC were selected. Age, the International Federation of Gynaecology and Obstetrics stage, gross pattern, histological grade, endometrioid intraepithelial neoplasia, adjacent or non-adjacent endometriosis or adenomyosis, and lymphovascular space invasion were assessed. Clinical factors were obtained from medical records.

### **EC of the Cancer Genome Atlas (TCGA)**

To compare survival curves with uMLAs, we used clinical data of 232 ECs, which were classified as four integrated clusters, namely *POLE* (n = 17), MSI (n = 65), copy-number low (n = 90), and copy-number high (n = 60), of TCGA. [9]

## **Results**

## **Genetic differences between oMLA and other histotypes**

We conducted WES for the following four ovarian cases with distinct histotypes: oMLA, endometrioid tumor (benign-borderline), OEC, and clear cell carcinoma (CCC) (Case 22); oMLA and OEC (Case 36); oMLA and HG component (Case 17); and non-MLA (OEC) and HG component (Case 37). Online Resource Figure 9 displays representative components of Cases 17 and 22. All components exhibited *KRAS* mutations in codon 12 (Online Resource Tables 7–10 and Online Resource Figure 14). In Case 22, *PIK3CA* p.H1047R and *CTNNB1* p.S37F mutations occurred solely in the oMLA and OEC. Copy number variations (CNVs) of oMLA were prominent and only the oMLA component showed a gain of 12p12.1, where *KRAS* was located. These genetic variations supported a phylogenetic tree, as illustrated in Online Resource Figure 15. Similarly, in Case 36, CNVs were more conspicuous in oMLA than in OEC (Online Resource Figure 12). In inter-case comparisons, the CNVs of oMLA were more prominent than those of OEC, although both obtained CNVs as HG components (Case 17 vs Case 37, Online Resource Figures 7 and 10, respectively).

In oMLAs, gains of chromosomes 1q, 2, 10, and 12 and the loss of 1q were observed. Moreover, acceleration of CNVs in oMLA was observed in Cases 22 (oMLA vs OEC vs CCC) and 36 (oMLA vs OEC).

**References**

1.	Djordjevic B, Hennessy BT, Li J, Barkoh BA, Luthra R, Mills GB, Broaddus RR (2012) Clinical assessment of PTEN loss in endometrial carcinoma: immunohistochemistry outperforms gene sequencing. Modern Pathology 25:699-708. doi: 10.1038/modpathol.2011.208

2.	Guan B, Mao TL, Panuganti PK, Kuhn E, Kurman RJ, Maeda D, Chen E, Jeng YM, Wang TL, Shih Ie M (2011) Mutation and loss of expression of ARID1A in uterine low-grade endometrioid carcinoma. Am J Surg Pathol 35:625-632. doi: 10.1097/PAS.0b013e318212782a

3.	Yano M, Ito K, Yabuno A, Ogane N, Katoh T, Miyazawa M, Miyazawa M, Hasegawa K, Narahara H, Yasuda M (2019) Impact of TP53 immunohistochemistry on the histological grading system for endometrial endometrioid carcinoma. Modern Pathology 32:1023-1031. doi: 10.1038/s41379-019-0220-1

4.	Buhard O, Cattaneo F, Wong YF, Yim SF, Friedman E, Flejou JF, Duval A, Hamelin R (2006) Multipopulation analysis of polymorphisms in five mononucleotide repeats used to determine the microsatellite instability status of human tumors. J Clin Oncol 24:241-251. doi: 10.1200/JCO.2005.02.7227

5.	Hall G, Clarkson A, Shi A, Langford E, Leung H, Eckstein RP, Gill AJ (2010) Immunohistochemistry for PMS2 and MSH6 alone can replace a four antibody panel for mismatch repair deficiency screening in colorectal adenocarcinoma. Pathology 42:409-413. doi: 10.3109/00313025.2010.493871

6.	McKenna A, Hanna M, Banks E, Sivachenko A, Cibulskis K, Kernytsky A, Garimella K, Altshuler D, Gabriel S, Daly M, DePristo MA (2010) The Genome Analysis Toolkit: a MapReduce framework for analyzing next-generation DNA sequencing data. Genome Res 20:1297-1303. doi: 10.1101/gr.107524.110

7.	Wang K, Li M, Hakonarson H (2010) ANNOVAR: functional annotation of genetic variants from high-throughput sequencing data. Nucleic Acids Res 38:e164. doi: 10.1093/nar/gkq603

8.	Soong D, Stratford J, Avet-Loiseau H, Bahlis N, Davies F, Dispenzieri A, Sasser AK, Schecter JM, Qi M, Brown C, Jones W, Keats JJ, Auclair D, Chiu C, Powers J, Schaffer M (2020) CNV Radar: an improved method for somatic copy number alteration characterization in oncology. BMC Bioinformatics 21:98. doi: 10.1186/s12859-020-3397-x

9.	Cancer Genome Atlas Research N, Kandoth C, Schultz N, Cherniack AD, Akbani R, Liu Y, Shen H, Robertson AG, Pashtan I, Shen R, Benz CC, Yau C, Laird PW, Ding L, Zhang W, Mills GB, Kucherlapati R, Mardis ER, Levine DA (2013) Integrated genomic characterization of endometrial carcinoma. Nature 497:67-73. doi: 10.1038/nature12113

# Online Resource Table 1. Primary antibodies used in immunohistochemistry

| Name | Manufacturer | Clone | Dilution |
| --- | --- | --- | --- |
| ARID1A | Abcam, Cambridge, UK | EPR13501-73 | 1:1000 |
| Calretinin | Roche Diagnostics, Arizona, USA | SP65 | Prediluted |
| CD10 | Leica Biosystems, Illinois, USA | 56C6 | 1:40 |
| ER | Roche Diagnostics, Arizona, USA | SP1 | Prediluted |
| GATA3 | Nichirei Biosciences, Tokyo, JPN | L50-823 | Prediluted |
| TP53 | Agilent, California, USA | DO-7 | 1:50 |
| PR | Roche Diagnostics, Arizona, USA | 1E2 | Prediluted |
| PTEN | Roche Diagnostics, Arizona, USA | SP218 | Prediluted |
| Thyroglobulin | Agilent, California, USA | DAK-Tg6 | 1:50 |
| TTF1 | Agilent, California, USA | 8G7G3/1 | 1:50 |
| WT1 | Agilent, California, USA | 6FH2 | Prediluted |

# Online Resource Table 2. Primer sequences targeted for *KRAS* hotspot mutations

| Gene | Targeted Codon | Exon | Sequence | Length (bp) |
| --- | --- | --- | --- | --- |
| *KRAS* | 12 | 2 | Forward: 5'-AAAAGGTACTGGTGGAGTATTTGA-3' | 293 |
|  |  |  | Reverse: 5'-TCATGAAAATGGTCAGAGAAACC-3' |  |

# Online Resource Table 3. Pathological characteristics in sfavoring vs indeterminate mesonephric-like adenocarcinoma (MLA) morphology, typical vs atypical immunohistochemistry (IHC), and MLA vs non-MLA

|  |  |  | Favoring vs Indeterminate morphology | | |  | Typical vs Atypical IHC | | |  | MLA vs non-MLA | | |
| --- | --- | --- | --- | --- | --- | --- | --- | --- | --- | --- | --- | --- | --- |
|  | All cases |  | Confirmed | Suspected | *P*-value |  | Typical | Atypical | *P*-value |  | MLA | non-MLA | *P*-value |
| Cases | 83 |  | 44/83 (53%) | 39/83 (47%) | N/A |  | 75/83 (90%) | 8 (10%) | N/A |  | 78/83 (94%) | 5/83 (6%) | N/A |
| Organ (uterus) | 47 (57%) |  | 25/44 (57%) | 22/39 (56%) | N/A |  | 44/75 (59%) | 3/8 (38%) | N/A |  | 47/78 (60%) | 0/5 (0%) | 0.013 |
| Favoring MLA morphology | 44 (53%) |  | N/A | | |  | 40/75 (53%) | 4/8 (50%) | N.S. |  | N/A | | |
| Predominant Architecture |  |  |  |  |  |  |  |  |  |  |  |  |  |
| Ductal and papillary | 26 (31%) |  | 4 (9%) | 22 (56%) | < 0.0001 |  | 23 (31%) | 3 (38%) | N.S. |  | 23 (30%) | 3 (60%) | N.S. |
| Tubulocystic | 1 (1%) |  | 1 (2%) | 0 (0%) | N.S. |  | 1 (1%) | 0 (0% |  |  | 1 (1%) | 0 (0%) |  |
| Micropapillary | 4 (5%) |  | 0 (0%) | 4 (10%) | 0.045 |  | 4 (5%) | 0 (0%) |  |  | 4 (5%) | 0 (0%) |  |
| Tubular | 30 (36%) |  | 21 (48%) | 9 (23%) | 0.024 |  | 28 (37%) | 2 (25%) |  |  | 29 (37%) | 1 (20%) |  |
| Spindled | 6 (7%) |  | 6 (14%) | 0 (0%) | 0.027 |  | 5 (7%) | 1 (13%) |  |  | 6 (8%) | 0 (0%) |  |
| Glomeruloid | 1 (1%) |  | 1 (2%) | 0 (0%) | N.S. |  | 1 (1%) | 0 (0%) |  |  | 1 (1%) | 0 (0%) |  |
| Labyrinthine | 5 (6%) |  | 3 (7%) | 2 (5%) | N.S. |  | 4 (5%) | 1 (12%) |  |  | 4 (5%) | 1 (20%) |  |
| Solid | 10 (2%) |  | 8 (18%) | 2 (5%) | N.S. |  | 9 (12%) | 1 (13%) |  |  | 10 (13%) | 0 (0%) |  |
| Fine nuclear pattern | 66 (80%) |  | 39 (89%) | 27 (69%) | 0.033 |  | 59 (79%) | 7 (88%) |  |  | 62 (80%) | 4 (80%) |  |
| High N/C ratio | 68 (82%) |  | 42 (95%) | 26 (67%) | 0.0011 |  | 61 (81%) | 7 (88%) |  |  | 64 (82%) | 4 (80%) |  |
| Squamous or mucinous metaplasia | 1 (1%) |  | 0 (0%) | 1 (3%) | N.S. |  | 1 (1%) | 0 (0%) |  |  | 1 (1%) | 0 (0%) |  |
| Large pale cytoplasmic nest | 36 (43%) |  | 21 (48%) | 15 (38%) | N.S. |  | 34 (45%) | 2 (25%) |  |  | 36 (46%) | 0 (0%) |  |
| Hobnail-like cells | 19 (23%) |  | 7 (16%) | 12 (31%) | N.S. |  | 18 (24%) | 1 (13%) |  |  | 18 (23%) | 1 (20%) |  |
| IHC |  |  |  |  |  |  |  |  |  |  |  |  |  |
| TTF1 (%) (IQR) | 20 (1–50) |  | 7.5 (0–50) | 20 (2–50) | N.S. |  | N/A | | |  | 20 (1–50) | 20 (2–45) | N.S. |
| GATA3 (%) (IQR) | 13 (3–30) |  | 15 (3.5–30) | 8 (1–30) | N.S. |  | N/A | | |  | 13 (3–30) | 0 (0–4) | 0.006 |
| CD10 (%) (IQR) | 5 (1–23) |  | 5 (0–30) | 5 (1–20) | N.S. |  | 5 (1 – 20) | 2.5 (0 – 9) | N.S. |  | 5 (1–23) | 0 (0–2.5) | 0.015 |
| Calretinin (%) (IQR) | 5 (1–10) |  | 5 (1–19) | 3 (0–5) | N.S. |  | 5 (1 – 10) | 1 (0 – 12) | N.S. |  | 5 (1–10) | 0 (0–8) | N.S. |
| ER (%) (IQR) | 0 (0–0) |  | 0 (0–0) | 0 (0–0) | N.S. |  | N/A | | |  | 0 (0–0) | 10 (0–80) | 0.0029 |
| *KRAS* exon 2 codon 12 mutation | 70 (84%) |  | 35 (80%) | 35 (90%) | N.S. |  | 64 (85%) | 6 (75%) | N.S. |  | 66 (84%) | 4 (80%) | N.S. |

N/C, nuclear to cytoplasmic ratio; IHC, immunohistochemistry; IQR, interquartile range; MLA, mesonephric-like adenocarcinoma; N/A, not assessed; N.S., not significan

# Online Resource Table 5. Comparison of ovarian mesonephric-like adenocarcinoma (oMLA) and ovarian endometrioid carcinoma (OEC)

|  | oMLA | OEC | *P* -value |
| --- | --- | --- | --- |
|  | (n = 31) | (n = 100) |  |
| Age (years old) (IQR) | 63 (58–72) | 57 (48–67) | 0.031 |
| FIGO Stage 2014 |  |  |  |
| I | 22 (71%) | 78 (78%) | 0.63  (I vs  II–IV) |
| II | 6 (19%) | 14 (14%) |  |
| III | 0 (0%) | 5 (5%) |  |
| IV | 3 (10%) | 3 (3%) |  |
| Solid predominant growth | 20 (65%) | 51 (51%) | 0.22 |
| Histological grade |  |  |  |
| 1 | N/A | 64 (64%) | N/A |
| 2 |  | 29 (29%) |  |
| 3 |  | 7 (7%) |  |
| LVSI | 7 (23%) | 10 (10%) | 0.12 |
| Endometriosis | 29 (94%) | 93 (93%) | 1.00 |

OEC, ovarian endometrioid carcinoma; FIGO, the International Federation of Gynecology and Obstetrics; IQR, interquartile range; LVSI, lymphovascular space invasion; N/A, not assessed; oMLA, ovarian mesonephric-like adenocarcinoma.

# Online Resource Table 7. Exonic functional gene variants between ovarian mesonephric-like adenocarcinoma (oMLA) and high-grade (HG) components by whole-exome sequencing, Case 17

| Case 17 |  |  |  | oMLA | | | HG component | | |
| --- | --- | --- | --- | --- | --- | --- | --- | --- | --- |
| Gene symbol | ExonicFunc.refGene | Nucleotide Change | Aa change | RefSeq Count | AltSeq Count | VAF | RefSeq Count | AltSeq Count | VAF |
| *KRAS* | nonsynonymous SNV | NM_001369786:c.35G>C | p.G12A | 38 | 91 | 71 | 61 | 138 | 69 |
| *RAF1* | nonsynonymous SNV | NM_001354695:c.1495C>G | p.L499V | 54 | 13 | 19 | 75 | 26 | 26 |

MLA, mesonephric-like adenocarcinoma; HG, high-grade; SNV, single-nucleotide variant; VAF, variant allele frequency.

# Online Resource Table 8. Exonic functional gene mutations in different components by whole-exome sequencing, Case 22

| Case 22 |  |  |  | BET | | | oMLA | | | OEC | | | CCC | | |
| --- | --- | --- | --- | --- | --- | --- | --- | --- | --- | --- | --- | --- | --- | --- | --- |
| Gene symbol | ExonicFunc.refGene | Nucleotide Change | Aa change | RefSeq Count | AltSeq Count | VAF | RefSeq Count | AltSeq Count | VAF | RefSeq Count | AltSeq Count | VAF | RefSeq Count | AltSeq Count | VAF |
| *KRAS* | nonsynonymous SNV | NM_001369786:c.35G>T | p.G12V | 34 | 29 | 46 | 1 | 23 | 96 | 14 | 14 | 50 | 37 | 13 | 26 |
| *PIK3CA* | nonsynonymous SNV | NM_006218:c.3140A>G | p.H1047R | 45 | 10 | 18 | 13 | 8 | 38 | 16 | 9 | 36 | 22 | 4 | 15 |
| *CTNNB1* | nonsynonymous SNV | NM_001098209:c.110C>T | p.S37F | 57 | 0 | 0 | 11 | 17 | 61 | 25 | 14 | 36 | 19 | 0 | 0 |

BET, benign endometrioid tumour; CCC, clear cell carcinoma; OEC, ovarian endometrioid carcinoma; oMLA, ovarian mesonephric-like adenocarcinoma; SNV, single-nucleotide variant; VAF, variant allele frequency.

# Online Resource Table 9. *KRAS* mutation between ovarian mesonephric-like adenocarcinoma (oMLA) and endometrioid ovarian carcinoma (OEC) components by whole-exome sequencing, Case 36

| Case 36 |  |  |  | oMLA | | | OEC | | |
| --- | --- | --- | --- | --- | --- | --- | --- | --- | --- |
| Gene symbol | ExonicFunc.refGene | Nucleotide Change | Aa change | RefSeq Count | AltSeq Count | VAF | RefSeq Count | AltSeq Count | VAF |
| *KRAS* | nonsynonymous SNV | NM_001369786:c.35G>A | p.G12D | 51 | 29 | 36 | 101 | 57 | 36 |

OEC, ovarian endometrioid carcinoma; oMLA, ovarian mesonephric-like adenocarcinoma; SNV, single-nucleotide variant; VAF, variant allele frequency.

# Online Resource Table 10. Exonic functional gene mutations between ovarian endometrioid carcinoma (OEC) with mesonephric-like differentiation (MLD) and high-grade (HG) components by whole-exome sequencing, Case 37

| Case 37 |  |  |  | OEC with MLD | | | HG component | | |
| --- | --- | --- | --- | --- | --- | --- | --- | --- | --- |
| Gene symbol | ExonicFunc.refGene | Nucleotide Change | Aa change | RefSeq Count | AltSeq Count | VAF | RefSeq Count | AltSeq Count | VAF |
| *KRAS* | nonsynonymous SNV | NM_001369786:c.35G>T | p.G12V | 21 | 18 | 46 | 76 | 97 | 56 |
| *SMARCA4* | frameshift insertion | NM_001128845:c.3354_3355insCG | p.G1120Afs*21 | 0 | 37 | 0 | 21 | 82 | 80 |

OEC, ovarian endometioid carcinoma; HG, high-grade; MLD, mesonephric-like differentiation; SNV, single-nucleotide variant; UC, undifferentiated carcinoma; VAF, variant allele frequency.

# Online Resource Figure 1. Architectural examples

Architectures depicted include ductal and papillary (a), tubulocystic (b), micropapillary (c), tubular (d), solid (e), glomeruloid (f), labyrinthine (g), cribriform (h), and spindled (i). In g, the labyrinthine architecture is characterized by a stromal core containing vascular features (g, arrowhead) and tubules that are juxtaposed but do not agglutinate, in contrast to the cribriform architecture. In i, the spindled architecture features spindle tumor cells that are epithelioid rather than sarcomatoid, with small tubules frequently incorporated (i, arrowhead).

# Online Resource Figure 2. Flowchart of the speculated origin based on the adjacent preexisting lesion and tumor location in uterine mesonephric-like adenocarcinoma (uMLA)

*KRAS* hotspot mutation analysis through Sanger sequencing was performed to determine the morphologically speculated origin. The morphologically speculated origin with the *KRAS* hotspot mutation was considered a molecularly speculated origin.

E, endometrium; EIN, endometrioid intraepithelial neoplasia; EP, endometrial polyp; M, myometrium; uMLA, uterine mesonephric-like adenocarcinoma.

# Online Resource Figure 3. Non-mesonephric-like adenocarcinomas (non-MLAs) of the ovary presenting indeterminate MLA morphology and atypical immunohistochemistry

a, Ovarian endometrioid carcinoma (OEC) (Case 16).

Tumor cells with sex cord-like change formed tubular, ductal, and trabecular architecture.

b, OEC (Case 37). Tumor cells with a low nuclear to cytoplasmic ratio formed tubular and ductal architecture.

c, OEC (Case 46). Tumor cells with oval to angular nuclei formed tubular, ductal, and tubulocystic architecture.

d, High-grade serous carcinoma (Case 15). Tumor cells with coarse chromatin and prominent mitotic figures exhibited tubular and labyrinthine architecture. This case demonstrated a null pattern of p53 expression and harbored serous tubal intraepithelial carcinoma.

# Online Resource Figure 4. Survival curve of uterine mesonephric-like adenocarcinoma (uMLA) and endometrioid endometrial carcinoma (EEC)

a, Overall survival (OS) was not different between uMLA and EEC in the International Federation of Gynecology and Obstetrics (FIGO) stage I. b, OS was not different between uMLA and EEC in FIGO stage II–IV. c, Progression-free survival (PFS) of uMLA was worse than that of EEC in FIGO stage I–II. d, PFS was not different between uMLA and EEC in FIGO stage III–IV.

# Online Resource Figure 5. Survival curve of uterine mesonephric-like adenocarcinoma (uMLA) and molecular subtypes of endometrial carcinoma.

a, Progression-free survival of uMLA was comparable to that of high copy-number (CN). b, Overall survival of uMLA was also comparable to that of high CN

MSI, microsatellite instability.

# Online Resource Figure 6. Survival curve of ovarian mesonephric-like adenocarcinoma (oMLA) and ovarian endometrioid carcinoma (OEC)

a, Overall survival (OS) did not differ between oMLA and OEC in the International Federation of Gynecology and Obstetrics (FIGO) stage I. b, OS of oMLA was worse than that of OEC in FIGO II–IV. c, d, Progression-free survival was not different between oMLA and OEC in early (FIGO I–II) vs advanced (FIGO II–IV) stages.

# Online Resource Figure 7. Overall survival (OS) curve of 22 pure ovarian mesonephric-like adenocarcinoma (oMLA) without other histotypes and ovarian endometrioid carcinoma (OEC)

a, Progression-free survival (PFS) was not different between pure oMLA and OEC.

b, Overall survival was not different between pure oMLA and OEC.

c, PFS was not different between pure oMLA and OEC in the International Federation of Gynecology and Obstetrics (FIGO) stage I.

d, PFS of pure oMLA was worse than that of OEC in FIGO stage II–IV.

# Online Resource Figure 8. Uterine mesonephric-like adenocarcinomas (uMLAs) with molecularly speculated origins

a-c depicted with adjacent adenomyosis (Case 63).

a, endophytic tumor confined to the myometrium. The endometrium was smooth without the involvement of a tumor.

b, adjacent adenomyosis and uMLA (arrowhead).

c, ER expressed in adenomyosis (arrowhead) and stromal cells but not in the uMLA. *KRAS* G12D mutation was identified in both the uMLA and adjacent adenomyosis. Therefore, adenomyosis was considered to be a molecularly speculated origin.

d and e uMLA with endometrioid intraepithelial neoplasm (EIN) (Case 21).

d, exophytic tumor, predominantly in the endometrium.

e, atypical endometrial cells exhibiting dense ductal architecture, indicative of EIN, were seen.

f, uMLA. tumor cells with fine chromatin and a high nuclear-to-cytoplasmic ratio forming tubule and ductal architecture indicative of MLA with an endometrioid carcinoma pattern. *KRAS* p.G12V mutation was identified in both the uMLA and EIN. Therefore, EIN was a molecularly speculated origin.

# Online Resource Figure 9. Ovarian mesonephric-like adenocarcinomas (oMLAs) and coexisting other histotypes, for which whole-exome sequencing was performed Case 17, oMLA component (a) and high-grade component (b).

Case 22, oMLA (c), ovarian endometrioid carcinoma (d), and clear cell carcinoma (e). Benign endometrioid tumor was seen (f).

# Online Resource Figure 10. Graphical plots of relative read depth and variant allele frequency across the whole exon, Case 17

a, mesonephric-like adenocarcinoma. b, high-grade component.

# Online Resource Figure 11. Graphical plots of relative read depth and variant allele frequency across the whole exon, Case 22

a, mesonephric-like adenocarcinoma. b, clear cell carcinoma. c, ovarian endometrioid carcinoma. d, endometrioid benign–borderline tumor.

# Online Resource Figure 12. Graphical plots of relative read depth and variant allele frequency across the whole exon, Case 36

a, mesonephric-like adenocarcinoma. b, ovarian endometrioid carcinoma.

# Online Resource Figure 13. Graphical plots of relative read depth and variant allele frequency across the whole exon, Case 37

a, Ovarian endometrioid carcinoma. b, high-grade component.

# Online Resource Figure 14. Overview of single-nucleotide variant (SNV) in *KRAS* codon 12 by whole-exon sequencing.

Every carcinoma component had SNVs in *KRAS*. VAF: variant allele frequency of *KRAS* codon 12. The pole color represents the kind of SNV with VAF: blue indicates wildtype, orange indicates c.35G>C, green indicates c.35G>T, and red indicates c.35G>A. Amp meant presence (+) or absence (-) of *KRAS* amplification; gain of chromosome 12p12.1, where *KRAS* is coded.

HG, high-grade components; oMLA, ovarian mesonephric-like adenocarcinoma; CCC, clear cell carcinoma; OEC, ovarian endometrioid carcinoma; VAF, variant allele frequency

# Online Resource Figure 15. Phylogenetic tree of Case 22

First, endometriosis with *KRAS* mutation developed into benign tumors and clear cell carcinoma (CCC). Second, *CTNNB1* and *PIK3CA* mutations occurred. Additionally, the tumor developed into ovarian endometrioid carcinoma (OEC). Meanwhile, copy number variations (CNVs) accelerated, and the tumor obtained the phenotype of ovarian mesonephric-like adenocarcinoma (oMLA).
